# Small events, big impact: Can rural sports event IP generate county-level tourism dividends? Evidence from “Village BA” in Taijiang County, China

**DOI:** 10.1371/journal.pone.0348851

**Published:** 2026-11-11

**Authors:** Jingmao Yan, Weifeng Guo, Yuanxin Liu, Yongjie Zhang, Minghan Cai, Huijie Guo

**Affiliations:** 1 School of Physical Education and Sport Science, Fujian Normal University, Fuzhou, Fujian, China; 2 College of Physical Education, Jimei University, Xiamen, Fujian, China; Linyi University, CHINA

## Abstract

Small-scale rural sports events are increasingly used as tools for regional regeneration, yet counterfactual evidence on whether they generate sustained tourism benefits at the county level remains limited. This study examines the rapid rise of “Village BA” in Taijing County, Guizhou, China, as a quasi-experimental setting. Using county-level panel data from 2015 to 2024, we estimate tourism responses associated with the post-2022 Village BA-centered expansion by combining the synthetic control method, synthetic difference-in-differences, and a matching-weighted two-way fixed-effects model. Across the baseline specifications, the estimated effects are positive for both outcomes, although their inferential profiles differ across estimators and robustness settings. First, the estimated effects in 2023–2024 are generally larger than those in the 2022 breakout stage, indicating a sustained post-breakout tourism response. Second, tourist-visitor estimates are comparatively more directionally stable across estimators and sensitivity specifications, whereas baseline tourism-revenue effects become more pronounced and more precisely estimated during the later institutional-expansion stage, a pattern broadly consistent with a relative “flows first, returns later” interpretation. SCM placebo rankings do not reach conventional significance levels, while SDID and matching-weighted TWFE provide alternative assessments of statistical precision. Sensitivity analyses further show that tourism-revenue estimates are more sensitive to estimator choice and the treatment of the COVID-affected pre-treatment years. These findings provide counterfactual evidence of county-level tourism gains associated with the Village BA-centered expansion, while the underlying mechanisms remain theory-grounded rather than directly identified.

## 1. Introduction

In global tourism governance and regional regeneration practice, sports events are increasingly understood not merely as competitive spectacles but as policy instruments for destination repositioning and economic growth [1,2]. Early event-economy research concentrated largely on mega-events such as the Olympic Games and the FIFA World Cup [3,4]. Yet a large body of work has repeatedly shown that such events are often accompanied by heavy infrastructure costs, post-event venue underutilization, and considerable leakage of returns through external supply chains and non-local ownership. Mega-events may also crowd out regular visitors through congestion, price inflation, and intensified security controls, thereby pushing destinations into a familiar “white elephant” dilemma of high cost and low return [5,6].

Against this backdrop, scholarship has undergone a clear conceptual shift—from short-run impact accounting to the strategic question of how event resources can be leveraged to create durable returns [7,8]. In line with this shift, rural and less-developed regions have increasingly explored a different path: the use of low-cost, community-led, high-frequency small-scale sports events as engines of endogenous development [9]. Compared with mega-events, small-scale events are more likely to rely on existing facilities and community organization, thereby reducing sunk costs and governance burdens while generating incremental demand through participant- and spectator-driven new consumption [10,11]. Event-specific studies have examined local economic benefits [12] and the motivations and involvement of amateur participants [13]. These studies provide useful insights into particular events and participants; the present study complements them by examining county-level tourism outcomes relative to an estimated counterfactual. Against this background, this study asks whether the tourism response to a small-scale rural sports event is short-lived or persists beyond its initial publicity surge.

China’s rural revitalization strategy offers a distinctive quasi-experimental setting for addressing this question. In recent years, the “Village BA” basketball tournament in Taipan Village, Taijing County, Guizhou, has rapidly gone viral, forming a new type of rural event IP grounded in community self-organization, Miao intangible cultural heritage, and social-media diffusion. Although the phenomenon has been discussed as both a model of rural sports rooted in local play culture and an instance of platform ruralism [14,15], a critical attribution problem remains unresolved: did the narrative of “heat” surrounding Village BA actually translate into county-level economic “retention”? And if so, was this due to the event itself, or merely a reflection of broader pandemic-related tourism disruptions, subsequent recovery, and macroeconomic trends?

Identifying a credible causal effect is challenging because the rise of event attention tends to co-occur with infrastructure improvements, destination marketing spillovers, and macroeconomic recovery. Without a rigorous counterfactual framework, common shocks can easily be mistaken for event-specific returns, reproducing the well-known tendency of traditional event-impact evaluations to overstate benefits [16]. To address this problem, this study treats Taijing County as a single treated unit and, using county-level panel data for 2015–2024, develops a complementary identification framework that combines SCM, SDID, and matching-weighted TWFE. SCM provides a data-driven synthetic Taijing as a transparent counterfactual benchmark [17]; SDID strengthens pre-treatment comparability through unit and time reweighting [18]; and matching-weighted TWFE serves as a parametric complement for stage-specific estimation of the 2022 breakout effect and the 2023–2024 post effect.

This study has three main contributions. First, it extends the empirical literature on rural small-scale sport events by providing, to our knowledge, the first county-level counterfactual evidence from a less-developed region of China. Second, it combines SCM, SDID, and matching-weighted TWFE in a unified design suited to a single-county shock, thereby improving identification relative to reliance on any single method. Third, it moves beyond a simple question of whether an effect exists and instead interprets the observed dynamics through the lenses of event leverage and affective spillover, thereby building an integrated framework that links counterfactual estimation, theory-grounded mechanism interpretation, and policy relevance.

## 2. Literature review

### 2.1 Sports events and regional tourism economies: from impact narratives to debates over net effects

Baade and Matheson’s review of the Olympic Games cautions that tourist spending and potential legacy benefits do not necessarily outweigh infrastructure and operating costs [4]. This highlights the need to distinguish event-related tourism activity from the net economic benefits accruing to host destinations. In response, sport-management scholarship has increasingly turned to the economic resilience of small-scale events. Compared with capital-intensive mega-events, small-scale events—because they rely on existing facilities and tend to attract stickier audiences—are more likely to generate endogenous circuits linking consumption, community, and industry, thereby reducing leakage and increasing the local retention of benefits [10,19]. In rural areas with weak industrial foundations, event-driven tourism development does not necessarily depend on major infrastructure shocks; instead, it can emerge through filling off-season gaps and stimulating participatory new consumption [20,21]. Even so, this proposition remains more conceptual than empirically verified at the macro level.

### 2.2 Micro-conversion mechanisms in sport–tourism integration: Spillovers from event experience to place attachment

Understanding the persistence of event-related tourism benefits requires attention to the psychological and behavioral mechanisms through which such effects may develop. Existing studies show that event experiences can substantially reshape tourists’ destination images and emotional ties [22]. In sport-tourism contexts, previous research has documented important links between event attachment and place attachment, providing a theoretical basis for understanding how attachment to an event may extend to the host destination [23]. For rural event IPs such as Village BA, which are deeply embedded in local culture, the combination of cultural authenticity and community participation may strengthen collective identification and emotional resonance. Such affective responses may extend visitors’ interest beyond the event itself and as suggested by prior research, strengthen revisit intentions and broader destination-related consumption, thereby providing a plausible micro-level foundation for the activation of event leverage [24]. In this sense, research on small-scale sport events suggests that they may avoid some of the structural drawbacks associated with mega-events while retaining considerable potential for tourism development and longer-term sustainability [25].

### 2.3 Methodological evolution in event evaluation: From input–output accounting to quasi-experimental identification

In the methodological evolution of event-impact evaluation, traditional multiplier and input–output approaches have been criticized for overstating benefits because they ignore substitution effects and resource constraints [16]. As a result, quasi-experimental methods grounded in the counterfactual framework have become increasingly central. SCM constructs a synthetic control through data-driven weighting, making it particularly well suited to single treated units; placebo and permutation procedures can then be used to strengthen inference [26]. Recent applications further show that SCM is capable of recovering credible counterfactual trajectories when a plausible control group is otherwise unavailable [27]. SDID, by combining reweighting with the difference-in-differences framework, can improve robustness when pre-treatment comparability is weak. For a single-county event shock such as Village BA, the joint use of SCM, SDID, and matching-weighted TWFE therefore provides a more credible basis for estimating tourism effects relative to constructed counterfactuals.

### 2.4 Research gaps and this study’s response

Despite the breadth of existing research on sports-event tourism, two major gaps remain in relation to emerging rural event IPs such as Village BA. The first is an explanation gap between micro mechanisms and macro performance. While psychological studies have documented the micro-level chain linking experience and attachment, these insights largely remain at the level of survey scales and perceived attitudes. Research on the symbolic meaning, cultural politics, and governance logic of Village BA is also growing, yet there is still no convincing macro-level evidence showing whether this “heat” was translated into sustainable county-level tourism dividends, and, if so, to what extent. The second is an identification gap. Although event evaluation has increasingly adopted quasi-experimental methods, conventional DID remains vulnerable to subjective control-group selection and weak trend comparability when the shock is highly specific and confined to a single county. In response, this study develops an integrated identification-and-explanation framework that uses SCM, SDID, and matching-weighted TWFE to estimate tourism responses associated with the post-2022 Village BA-centered expansion for both tourism revenue and tourist visitors and then interprets the resulting dynamics through the lens of potential event-leverage and consumption-conversion mechanisms.

## 3. Theoretical foundations and hypotheses

To explain how Village BA may generate county-level tourism effects, this study develops an integrated framework that departs from the standard mega-event lens and instead combines the theory of small-scale sport events, the rural sports and local games (RSLG) model, and event leverage theory.

### 3.1 Comparative advantage and economic resilience: The micro-logic of rural small-scale events

The supply-side logic of rural event dividends begins with the asset structure of small-scale events. The ‘theory of small-scale sports events’ proposed by Higham (1999) and Gibson et al. (2012) provides the foundational lens for this paper [10]. Unlike mega-events, which are highly capital dependent, small-scale events are characterized by light assets, reliance on existing facilities, and recurring frequency. In less-developed regions, these traits help avoid enclave-style development. Their comparative advantage lies, first, in low leakage and high local multiplier effects: because event organization depends heavily on local public services and community-based labor, event-induced consumption circulates within the community rather than flowing to external service providers [21]. Second, small-scale events can smooth seasonality by filling gaps in rural tourism demand and generating more stable cash flows. Village BA, in this sense, can be viewed as a locally embedded event model with the potential for greater economic resilience.

### 3.2 Cultural authenticity and attachment spillovers: The RSLG model in an endogenous-development perspective

From the demand side, the tourism appeal of Village BA can be understood through endogenous development theory, which emphasizes the mobilization of distinctive local cultural and social resources. The RSLG model proposed by Mousavi and colleagues provides an appropriate framework for interpreting this process [14]. Events rooted in local traditions—such as ethnic festivals and agrarian customs—derive an important part of their appeal from cultural authenticity, which is difficult to replicate elsewhere. In the case of Village BA, this cultural embeddedness may weaken the impersonal service–consumption relationship typical of highly commercialized events and instead foster a stronger sense of affective connection between hosts and visitors. Empirical work in psychology further suggests that high-involvement interaction of this kind may generate not only attachment to the event itself but also an “affection spillover” toward the host destination [23]. Such emotional translation therefore provides a theoretically plausible pathway through which event-related attention may contribute to more durable destination attachment and tourism retention.

### 3.3 Theoretical mechanism: Resource recombination under event leverage theory

To explain how event-generated attention may be translated into broader county-level tourism returns, this study draws on Chalip’s event leverage theory [1]. Unlike ex post impact accounting, leverage theory emphasizes the strategic recombination of destination resources before and during an event. In the case of Taijing, this theoretical logic can be conceptualized as a chain of IP-driven attraction, leverage amplification, and value capture. First, direct economic leverage may channel event-related demand toward food, accommodation, and other forms of local consumption. Second, destination-image leverage may use heightened media visibility to strengthen perceptions of Taijing as a vibrant, hospitable, and culturally distinctive destination, thereby helping to reshape pre-existing destination images. Third, cross-industry leverage may connect “sports+intangible cultural heritage” and “sports+agricultural specialties,” thereby directing event-related attention toward adjacent local industries [28]. Under this framework, growth in county-level tourism revenue need not be viewed as an automatic by-product of the event itself; rather, event leverage provides a theoretical explanation for how destination-level resource coordination may contribute to the conversion of event attention into broader tourism returns.

### 3.4 Hypotheses

Based on the foregoing theoretical logic, and especially on the low-leakage profile of small-scale events and the spillover effects emphasized in leverage theory, the study proposes the following hypotheses.

H1 (existence hypothesis): The 2022 social-media breakout and subsequent expansion of Village BA are expected to be associated with positive county-level tourism outcomes relative to Taijing’s counterfactual trajectory, after accounting for broader pandemic-related tourism disruptions and subsequent recovery.

H2 (dynamic hypothesis): The estimated tourism response is expected to be larger during the 2023–2024 institutional-expansion stage than during the immediate 2022 breakout stage. As event awareness diffuses and the Village BA-centered development process expands, event-related tourism responses may accumulate rather than dissipate immediately after the initial social-media breakout. Accordingly, the estimated tourism effects are expected to strengthen rather than fade as the event moves from initial breakout to institutional expansion.

H3 (outcome-difference hypothesis): The Village BA-centered expansion is expected to generate different temporal response patterns in tourist visitors and tourism revenue, reflecting the distinction between visitor attraction and the conversion of visitation into local spending.

## 4. Empirical strategy

### 4.1 Research context and identification challenges

Village BA originated in the traditional festival activities of Taipan Village in Taijing County, Guizhou. The event’s 2022 breakout phase was primarily driven by short-video diffusion and rapid growth in social attention, whereas the 2023 institutional-expansion phase corresponded to the institutionalized rollout of the national Hemei Rural Basketball Tournament under the joint organization of the Ministry of Agriculture and Rural Affairs and the General Administration of Sport. Rather than treating Village BA as a one-shot intervention, this study therefore distinguishes two stages: a 2022 breakout stage and a 2023–2024 institutional-expansion stage.

This setting generates three identification challenges. First, Taijing is a single treated unit, making standard multi-treated-group policy designs unsuitable. Second, both tourism revenue and tourist visitors are influenced by pandemic-related disruptions and subsequent recovery, macroeconomic fluctuations, fiscal input, and regional tourism policies, so common trends can easily be mistaken for event-related effects in the absence of a credible counterfactual. Third, the tourism response to Village BA is unlikely to be static; the relative responses of tourist visitors and tourism revenue may evolve differently across the breakout and institutional-expansion stages.

Importantly, these two stages are intended to distinguish the timing of the broader Village BA-centered development process rather than to separately identify the marginal effects of institutionalization, subsequent public support, or other contemporaneous interventions. Accordingly, the post-2022 estimates should be interpreted as the estimated tourism effects associated with a broader event-centered treatment bundle surrounding the expansion of Village BA, encompassing the tournament itself together with contemporaneous activities and support related to its development. To address these identification challenges, the study employs three complementary methods—SCM, SDID, and matching-weighted TWFE—to assess whether the direction and dynamic pattern of the estimated effects are consistent across alternative counterfactual and inferential frameworks.

### 4.2 Data and variables

The dataset is a multidimensional county–year panel covering the 16 county-level units of the Qiandongnan Miao and Dong Autonomous Prefecture, Guizhou, from 2015 to 2024. The main estimating sample is a balanced county–year panel with 160 observations.

Four early county–year cells contained tourism outcomes that were not directly tabulated in the available county-level statistical records: Taijing (2016), Zhenyuan (2017), Tianzhu (2016), and Majiang (2017). Because both tourism revenue and tourist visitors were affected in each case, these correspond to eight outcome observations. These observations were reconstructed using a deterministic growth-rate-based backcasting procedure. Backcasting is commonly used to reconstruct historical statistical series and maintain temporal consistency when earlier observations are unavailable [29]. Specifically, when the preceding-year value was unavailable but the subsequent-year level and the corresponding officially reported year-on-year growth rate were available, the value was recovered as


$$\begin{array}{c} \begin{array}{c} Y_{t-\text{1}}=\frac{Y_{t}}{\text{1}+g_{t}} \end{array} \end{array}$$ (1)

where $Y_{t}\;$ denotes the subsequent-year tourism outcome and $g_{t}\;$ denotes the officially reported year-on-year growth rate. Using this arithmetic relationship, the reconstructed values were RMB 0.800 billion and 1.220 million tourist visitors for Taijiang; RMB 8.560 billion and 11.200 million tourist visitors for Zhenyuan; RMB 1.020 billion and 1.450 million tourist visitors for Tianzhu; and RMB 1.261 billion and 2.135 million tourist visitors for Majiang.

The study uses two outcome variables to capture both the revenue and flow dimensions of tourism development: the natural logarithm of annual tourism revenue, $\ln\left({\text{TourismRevenue}}_{it}\right)$, and the natural logarithm of annual tourist visitors, $\text{ln}\left({\text{TouristVisitors}}_{it}\right)$. The former reflects the economic return to tourism, while the latter captures the scale of visitor inflows. Modeling these outcomes separately allows the study to compare how visitor flows and tourism revenue evolved relative to their respective counterfactual trajectories.

Two treatment interactions are constructed to reflect the staged nature of the shock: ${Treatment}_{i}\times \text{1}[year=\text{2022}]$,which captures the immediate breakout effect, and ${Treatment}_{i}\times \text{1}[year\geq \text{2023}]$,which captures the average post effect during the institutional-expansion stage. $Treatment_{i}$ equals 1 for Taijiang County and 0 otherwise.

Control variables include the logarithms of per-capita GDP, resident population, budget revenue, and budget expenditure. Per-capita GDP captures differences in local economic development, resident population reflects regional scale, and budget revenue and expenditure capture local fiscal and public-service capacity. Prior tourism research has highlighted the relevance of economic development and government expenditure to tourism demand, tourist flows, and tourism receipts [30,31]. These variables are included as covariates in the matching-weighted TWFE specification and as predictors in the SCM specification. The SDID models are estimated using the outcome, county, year, and treatment indicators without additional covariates. More tourism-specific indicators, such as accommodation capacity, transport accessibility, and tourism infrastructure, would also be theoretically relevant. However, consistently measured and comparable county-level series are not available for all sample counties over the full pre-treatment period.

Tourist visitors are not included as a control in the tourism-revenue equation. Because tourism revenue can be conceptually decomposed into visitor volume multiplied by per-capita spending, conditioning on tourist visitors would introduce a classic bad-control problem and attenuate estimation of the overall post-2022 treatment effect on tourism revenue. Tourist visitors are therefore modeled as a separate outcome. Variable definitions and data sources are summarized in Table 1.

**Table 1:** Variable definitions and data sources.

Variable Type	Variable Name	Indicator	Measurement	Data Source
Outcome variable	Tourism revenue	$\ln({TourismRevenue}_{it})$	Natural logarithm of annual tourism revenue in county-level unit i in year t (RMB 100 million)	Qiandongnan Statistical Yearbook; county/city statistical bulletins
	Tourist visitors	$\ln({TouristVisitors}_{it})$	Natural logarithm of annual tourist visitors (10,000 persons) in county-level unit i in year t	Qiandongnan Statistical Yearbook; county/city statistical bulletins
Core explanatory variable	Immediate effect in 2022	${Treatment}_{i}\times \text{1}[year=\text{2022}]$	Captures the Taijing-specific post-breakout contrast in 2022	General Administration of Sport of China; local government bulletins
	Post effect in 2023–2024	${Treatment}_{i}\times \text{1}[year\geq \text{2023}]$	Captures the average Taijing-specific post-treatment contrast during the institutional-expansion stage	General Administration of Sport of China; local government bulletins
Control variable	Level of economic development	$\ln\;\left({pcgdp}_{it}\right)$	Natural logarithm of annual per-capita GDP (RMB per person)	China County Statistical Yearbook
	Population size	$\ln{(Population}_{it})$	Natural logarithm of resident population (10,000 persons)	Qiandongnan Statistical Yearbook; county/city statistical bulletins
	Budget revenue	$\ln{(BudgetRevenue}_{it})$	Natural logarithm of general public budget revenue (RMB 10,000)	Qiandongnan Statistical Yearbook; county/city statistical bulletins
	Budget expenditure	$\ln({BudgetExpenditure}_{it})$	Natural logarithm of general public budget expenditure (RMB 10,000)	Qiandongnan Statistical Yearbook; county/city statistical bulletins

### 4.3 Methods

#### 4.3.1 Synthetic control method (SCM).

Given that Taijing constitutes a single treated unit, the study first applies the synthetic control method (SCM). SCM constructs a synthetic Taijing from untreated donor counties using non-negative weights chosen to minimize pre-treatment discrepancies in outcomes and predictors. The post-treatment difference between actual Taijing and synthetic Taijing is then interpreted as the estimated post-treatment gap for each period. Formally, SCM assigns non-negative donor weights that sum to one and uses the weighted combination of donor outcomes to construct Taijing’s counterfactual trajectory. The same SCM specification, predictor set, 2015–2021 fitting period, and 2022 treatment date are applied consistently in the baseline estimation and the in-space placebo analysis to ensure comparability across treatment assignments. Tourism revenue and tourist visitors are modeled separately as outcomes, with the SCM predictor set including the annual pre-treatment history of the respective tourism outcome, the alternative tourism outcome, and the covariates defined in Section 4.2. This study utilized Stata 17.0 for data analysis.

The predictor weights are determined endogenously from the pre-treatment data. Under the formal non-nested SCM specification, the estimated V-weights place nearly all relative predictive weight on the annual pre-treatment histories of the respective outcome variables, whereas the macroeconomic and fiscal covariates, as well as the alternative tourism outcome, receive zero or negligible weights at the reported precision. Thus, although these variables remain part of the predictor set, the construction of synthetic Taijing is driven primarily by similarity in historical tourism trajectories. The corresponding predictor V-weights are reported in S1 Table.

Baseline effects are reported separately for the 2022 breakout stage and the 2023–2024 institutional-expansion stage.

#### 4.3.2 Synthetic difference-in-differences (SDID).

To reduce reliance on the standard parallel-trends assumption and improve pre-treatment comparability under a single treated unit, the study further applies SDID. SDID combines the DID logic with unit and time reweighting so that treated and control units are made more comparable before treatment. Relative to SCM, SDID offers inference closer to a parametric DID framework; relative to conventional DID, it is better suited to weakly comparable control settings. It is therefore used as a complementary rather than redundant estimator. Statistical uncertainty for the SDID estimates is assessed using placebo-based variance estimation. Because the empirical setting contains a single treated county, we implement 1,000 placebo replications and report the resulting placebo-based standard errors, normal-approximation 95% confidence intervals, and p-values. These quantities provide an estimator-specific assessment of statistical precision for the SDID specification.

#### 4.3.3 Matching-weighted TWFE.

As a parametric complement, we estimate a matching-weighted two-way fixed-effects model with separate indicators for the 2022 breakout stage and the 2023–2024 institutional-expansion stage. Matching weights are constructed from eight standardized pre-treatment characteristics: the pre-treatment means and linear trends of log tourism revenue and log tourist visitors, together with the pre-treatment means of log per-capita GDP, log resident population, log budget revenue, and log budget expenditure.

Let di  denote the Euclidean distance between donor county i and Taijing in this eight-dimensional standardized pre-treatment feature space:


$$\begin{array}{c} \begin{array}{c} d_{i}={\left[\sum_{k=\text{1}}^{\text{8}}{\left(Z_{ik}-Z_{Tk}\right)}^{\text{2}}\right]}^{\text{1}/\text{2}} \end{array} \end{array}$$ (2)

where $Z_{ik}\;$ denotes donor county i’s standardized value for matching characteristic k and $Z_{Tk}\;$ denotes the corresponding standardized value for Taijiang. The unnormalized exponential-kernel weight is defined as


$$\begin{array}{c} \begin{array}{c} {\tilde{w}}_{i}=\text{exp}\left(-\lambda d_{i}\right) \end{array} \end{array}$$ (3)

with the decay parameter fixed at λ=1. The resulting donor weights are then normalized as


$$\begin{array}{c} \begin{array}{c} w_{i}=\frac{{\tilde{w}}_{i}}{\sum_{j\in D}{\tilde{w}}_{j}} \end{array} \end{array}$$ (4)

where D  denotes the donor pool, such that $\sum_{j\in D}w_{j}=\text{1}$. The weighting procedure reduces standardized pre-treatment differences across all included characteristics, although residual imbalance remains, particularly in per-capita GDP and resident population. We therefore interpret the procedure as improving, rather than eliminating, pre-treatment comparability. Detailed balance diagnostics are reported in S2 Table.

Because the matching weights are generated from the observed pre-treatment data, conventional second-stage standard errors condition on the estimated weights and do not incorporate uncertainty arising from weight construction. We therefore implement a full-procedure donor bootstrap with 1,000 replications. In each replication, the 15 donor counties are resampled with replacement at the county level, while Taijing remains the single treated unit. Each resampled donor contributes its full county-year trajectory. The pre-treatment features, standardization parameters, Euclidean distances, exponential-kernel weights, and second-stage TWFE coefficients are then recalculated in each replication. This procedure incorporates uncertainty arising from both donor composition and the generated matching weights. Given the small number of county clusters, we additionally conduct Webb wild-cluster bootstrap inference with 9,999 replications as a conservative finite sample check. Together, these procedures assess the statistical precision of the stage-specific TWFE estimates while accounting for generated-weight and small-cluster uncertainty.

### 4.4 Results

#### 4.4.1 Main results: Positive tourism responses associated with Village BA in Taijing County.

The baseline estimates are positive across SCM, SDID, and matching-weighted TWFE for both tourism revenue and tourist visitors, although the strength of statistical evidence differs across outcomes and inferential procedures. Overall, tourist-visitor responses show greater directional stability across sensitivity specifications, whereas tourism-revenue effects become larger during the later stage but remain more sensitive to estimator choice, inference method, and counterfactual construction.

For tourism revenue, SCM estimates a log gap of 0.262 in 2022 and an average gap of 0.664 in 2023–2024, corresponding to approximate proportional effects of 29.95% and 94.31%, respectively. SDID produces somewhat larger estimates. As reported in Table 2, the estimated ATT is 0.398 in 2022 (SE = 0.137, 95% CI [0.129, 0.667], p = 0.004) and 0.794 in 2023–2024 (SE = 0.155, 95% CI [0.490, 1.097], p < 0.001), corresponding to approximate increases of 48.87% and 121.14%.

**Table 2:** Baseline SDID estimates and placebo-based inference.

Outcome	Period	ATT	Placebo SE	95% CI	p-value	Effect (%)
Tourism revenue	2022	0.398	0.137	[0.129,0.667]	0.004	48.87
Tourism revenue	2023–2024	0.794	0.155	[0.490,1.097]	<0.001	121.14
Tourist visitors	2022	0.345	0.123	[0.104,0.587]	0.005	41.25
Tourist visitors	2023–2024	0.556	0.279	[0.009,1.103]	0.046	74.31

ATT denotes the SDID treatment effect. Standard errors are placebo-based using 1,000 replications. The 95% confidence intervals and p-values are based on the corresponding normal approximation using the placebo-based standard errors. Percentage effects are calculated as 100[exp(ATT)−1].

SDID provides a complementary estimator-specific assessment of the treatment response and its statistical precision. As shown in Table 2, placebo-based confidence intervals exclude zero for both outcomes and both treatment stages in the baseline specification. The later-stage visitor estimate retains a comparatively wide interval, whereas the later-stage revenue interval excludes zero by a clear margin. SDID is therefore interpreted as a complementary specification with its own placebo-based uncertainty assessment rather than merely as a directional check on SCM. Its results should be considered jointly with the SCM counterfactual gaps and the more conservative Webb wild-cluster results from the matching-weighted TWFE model discussed below.

To place these proportional estimates in absolute monetary terms, Taijing’s observed tourism revenue was RMB 1.712 billion in 2022, compared with RMB 1.317 billion for synthetic Taijing, implying an absolute gap of approximately RMB 0.395 billion. In 2023, observed and synthetic tourism revenue were RMB 3.333 billion and RMB 1.714 billion respectively, corresponding to an absolute gap of approximately RMB 1.619 billion. In 2024, the corresponding values were RMB 4.366 billion and RMB 2.248 billion, yielding an absolute gap of approximately RMB 2.118 billion. These absolute differences provide additional economic context for the relatively large proportional SCM estimates. Detailed annual monetary values are reported in S3 Table.

The matching-weighted TWFE estimates for tourism revenue are also positive but remain sensitive to the inferential procedure (Table 3). The estimated coefficient is 0.186 in 2022 and 0.516 in 2023–2024. When uncertainty from the generated matching weights is incorporated through the full-procedure bootstrap, the 95% confidence interval includes zero for the 2022 estimate [−0.084, 0.381] but excludes zero for the 2023–2024 estimate [0.230, 0.734]. Webb wild-cluster bootstrap inference is more conservative, yielding p-values of 0.320 and 0.054, respectively. Thus, the 2023–2024 revenue response is supported by the full-procedure bootstrap and approaches, but does not cross, the conventional 5% threshold under the more conservative small-cluster inference; the 2022 estimate remains imprecise.

**Table 3:** Matching-weighted TWFE estimates and alternative inference.

	(1) Log Tourism Revenue	(2) Log Tourist Visitors
Treatment × 2022	0.186	0.209
	(0.133)	(0.063)
	[−0.084, 0.381]	[0.051, 0.307]
Webb wild-cluster p-value	0.320	0.058
Treatment × Post (2023–2024)	0.516 (0.153)	0.457 (0.110)
	[0.230, 0.734]	[0.201, 0.614]
Webb wild-cluster p-value	0.054	0.030
ln(Per capita GDP)	0.490	0.505
	(0.372)	(0.448)
ln(Resident population)	1.538	1.474
	(1.843)	(1.752)
ln(Budget revenue)	−0.396	−0.089
	(0.315)	(0.119)
ln(Budget expenditure)	0.393	0.189
	(0.442)	(0.296)
R²	0.859	0.911
Adj. R²	0.826	0.890
N	160	160
County FE	Yes	Yes
Year FE	Yes	Yes
Controls	Yes	Yes

Conventional county-clustered standard errors are reported in parentheses. Full-procedure bootstrap 95% confidence intervals are reported in square brackets and are based on 1,000 replications, with the matching weights re-estimated in each replication. Webb wild-cluster p-values are based on 9,999 replications. All models include county and year fixed effects and the reported controls.

For tourist visitors, the evidence is comparatively more stable. SCM estimates a log gap of 0.229 in 2022 and an average gap of approximately 0.560 in 2023–2024, corresponding to approximate increases of 25.76% and 74.97%, respectively. The annual SCM gaps are 0.617 in 2023 and 0.502 in 2024, indicating a substantial post-treatment divergence in both years of the institutional-expansion stage. SDID yields an ATT of 0.345 in 2022 (SE = 0.123, 95% CI [0.104, 0.587], p = 0.005) and 0.556 in 2023–2024 (SE = 0.279, 95% CI [0.009, 1.103], p = 0.046), equivalent to approximate increases of 41.25% and 74.31%, respectively. Although the later-stage confidence interval is relatively wide, both baseline SDID estimates remain statistically distinguishable from zero.

The matching-weighted TWFE estimates for tourist visitors are 0.209 in 2022 and 0.457 in 2023–2024. Their full-procedure bootstrap 95% confidence intervals are [0.051, 0.307] and [0.201, 0.614], respectively, both excluding zero. Under the more conservative Webb wild-cluster bootstrap, the corresponding p-values are 0.058 and 0.030. These results provide marginal evidence of an immediate visitor-flow response in 2022 and stronger evidence of a sustained increase in 2023–2024.

Overall, the positive baseline estimates are broadly consistent with H1, although the strength of inferential support varies across outcomes and estimators. The three estimators provide complementary rather than statistically independent perspectives on the same underlying county panel. SCM documents the magnitude and evolution of the counterfactual gaps, SDID provides placebo-based uncertainty estimates under an alternative weighting structure, and matching-weighted TWFE provides stage-specific parametric estimates while accounting for generated-weight and small-cluster uncertainty. Across these specifications and inferential procedures, the results are more stable for tourist visitors than for tourism revenue. Revenue estimates remain positive in the baseline specifications but are more sensitive to estimator choice, inference procedure, and the construction of the pre-treatment counterfactual.

#### 4.4.2 Differences in the stability and evolution of the two tourism outcomes.

The two tourism outcomes display distinct response profiles across the two treatment stages. Tourist-visitor estimates are comparatively stable across the baseline estimators and inferential procedures, whereas tourism-revenue estimates are more sensitive to estimator choice and uncertainty correction. Baseline SDID estimates also show a positive tourism-revenue response in 2022, while the estimated effects for both outcomes increase during 2023–2024, with revenue effects becoming especially pronounced. The more pronounced tourism-revenue effects during the later institutional-expansion stage are broadly consistent with the temporal differentiation anticipated in H3. However, this descriptive pattern does not directly establish how visitor attractions are converted into local spending. Overall, this pattern broadly aligns with a relative “flows first, returns later” interpretation.

#### 4.4.3 SCM graphical evidence and finite-sample placebo inference.

Graphical evidence from SCM provides a transparent assessment of pre-treatment fit and post-treatment divergence for both tourism outcomes. As shown in Fig 1, actual Taijiang and synthetic Taijing follow broadly similar trajectories during the pre-treatment period for both tourism revenue and tourist visitors, supporting the credibility of the constructed counterfactuals. Following the 2022 breakout, the treatment gaps become positive for both outcomes and widen substantially during the 2023–2024 institutional-expansion stage (Fig 2). The in-space placebo paths (Fig 3) and the corresponding distributions of post/pre-RMSPE ratios (Fig 4) further show how Taijing’s post-treatment trajectories compare with those obtained when treatment is reassigned to the other counties.

**Fig 1:**
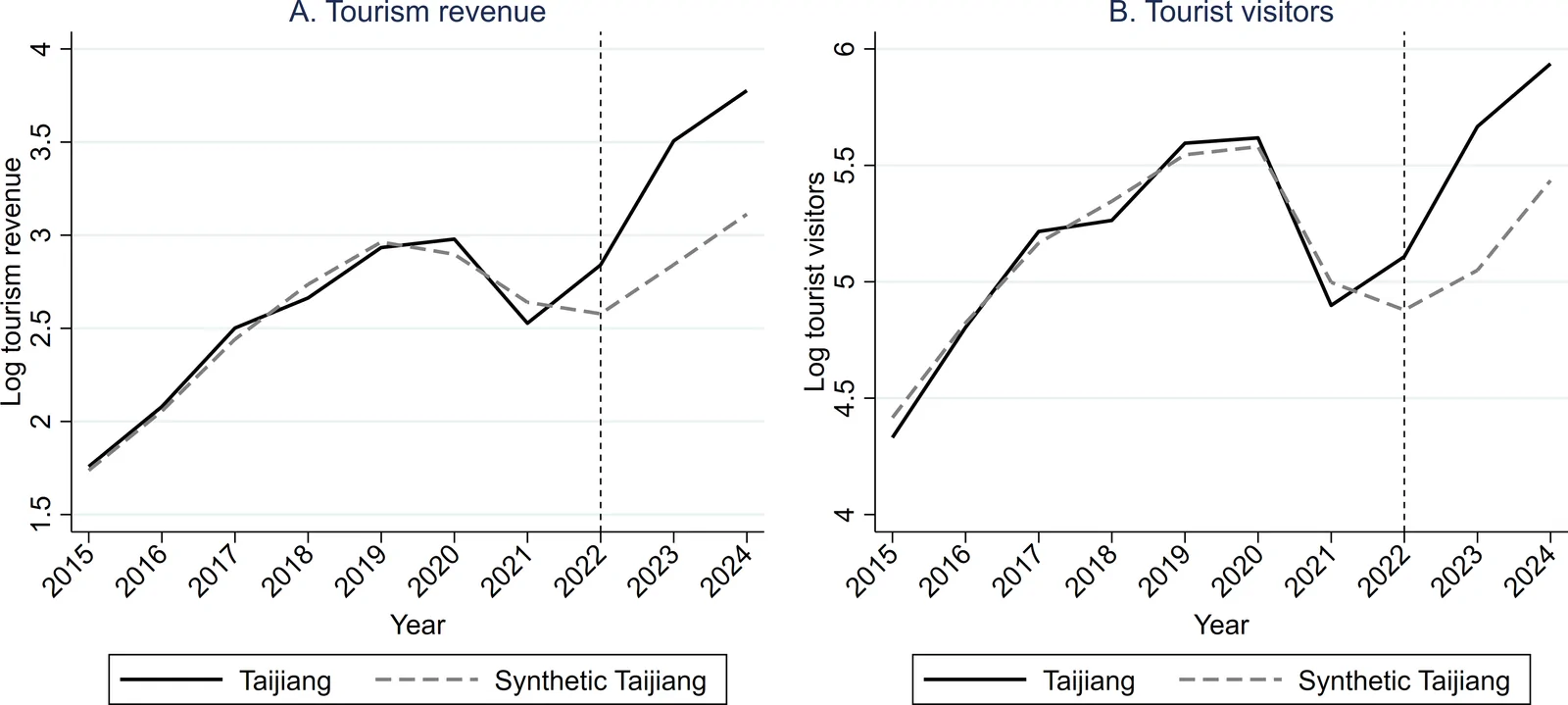
Actual and synthetic tourism trajectories in Taijing County.

**Fig 2:**
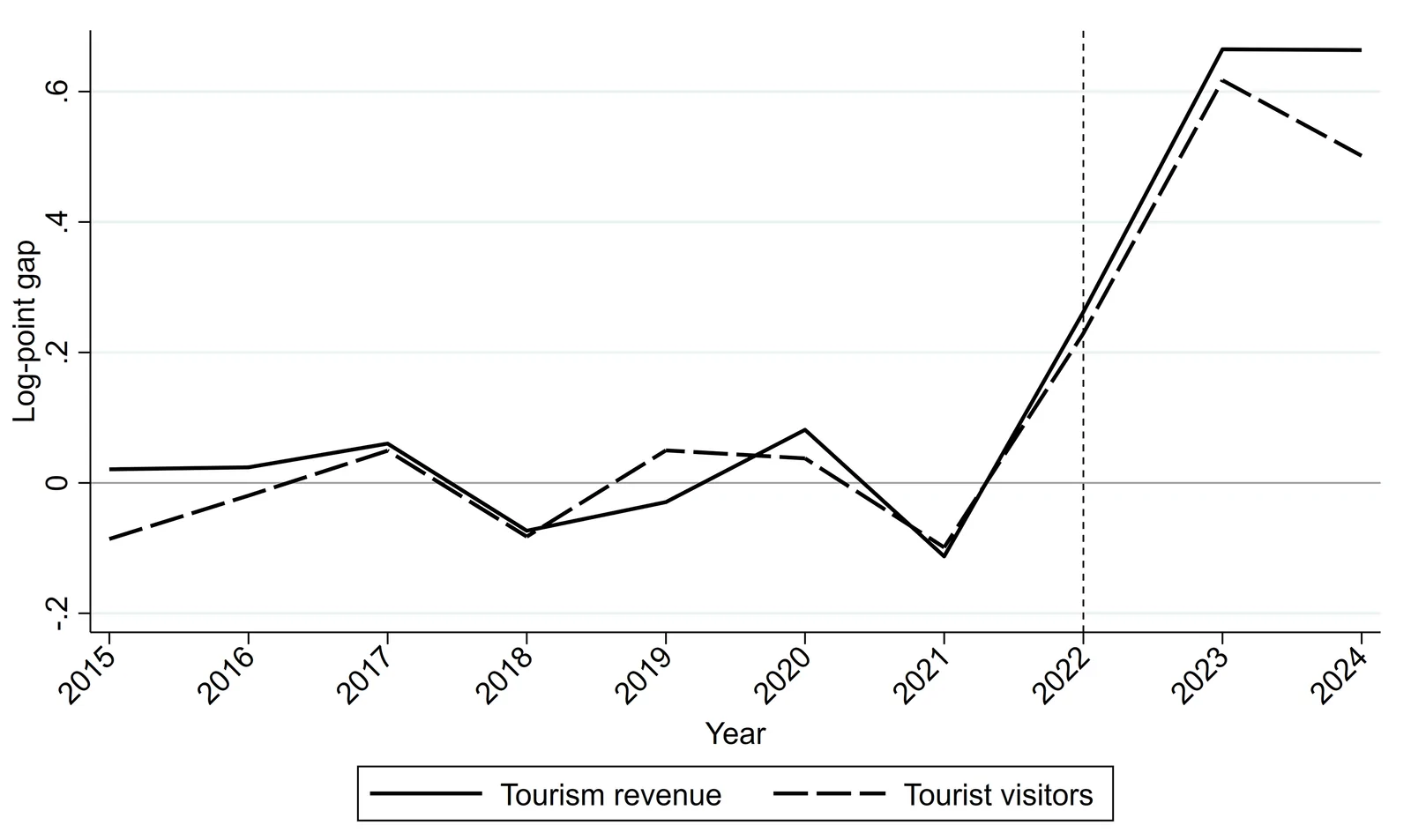
Synthetic-control treatment gaps for tourism revenue and tourist visitors.

**Fig 3:**
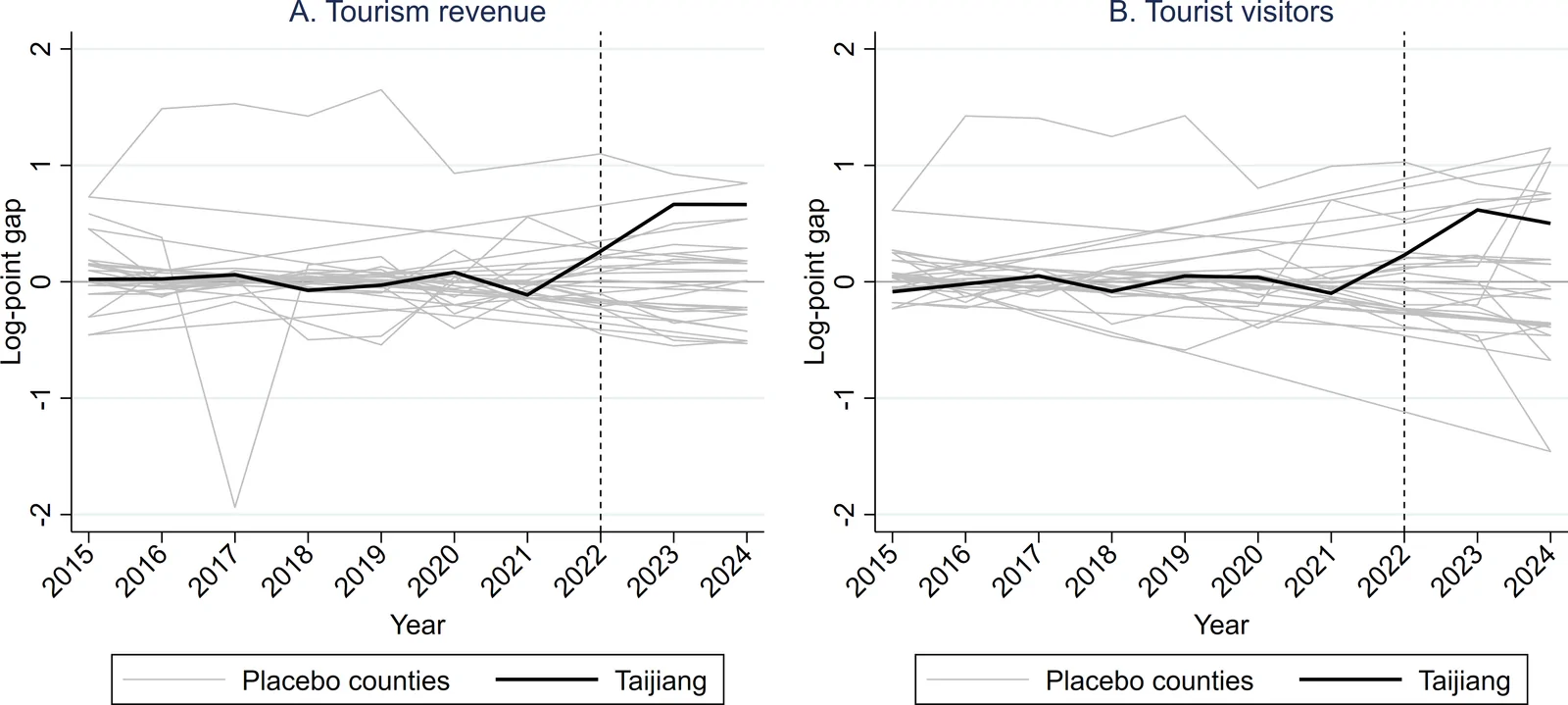
In-space placebo gap paths for tourism revenue and tourist visitors.

**Fig 4:**
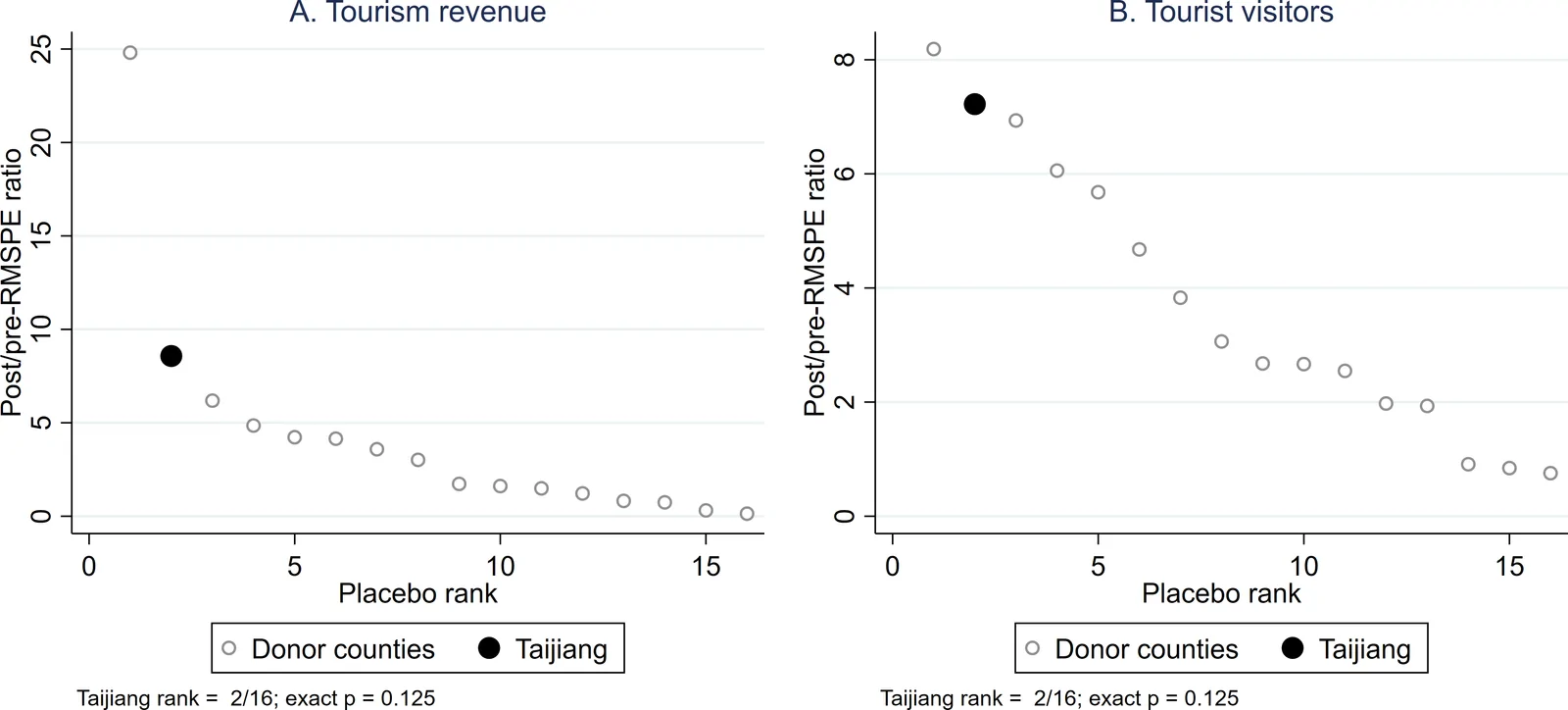
Post/pre-RMSPE ratios from the in-space placebo tests.

For the formal in-space placebo exercise, the same SCM specification, predictor set, 2015–2021 fitting period, and 2022 treatment date are applied uniformly to Taijing and all 15 placebo-treated counties. All 16 treatment assignments are successfully estimated. For tourism revenue, Taijing’s post/pre-RMSPE ratio is 8.574 and ranks second among the 16 assignments, yielding an exact in-space placebo p-value of


$$\begin{array}{c} \begin{array}{c} \text{P}=\frac{\text{2}}{\text{1}\text{6}}=\text{0}.\text{1}\text{2}\text{5} \end{array} \end{array}$$ (5)

For tourist visitors, Taijing’s post/pre-RMSPE ratio is 7.222 and ranks second among the 16 assignments, yielding


$$\begin{array}{c} \begin{array}{c} \text{P}=\frac{\text{2}}{\text{1}\text{6}}=\text{0}.\text{1}\text{2}\text{5} \end{array} \end{array}$$ (6)

Neither placebo result reaches conventional levels of statistical significance. Moreover, with only 16 possible treatment assignments, the smallest attainable one-sided exact p-value is


$$\begin{array}{c} \begin{array}{c} {\text{P}}_{min}=\frac{\text{1}}{\text{1}\text{6}}=\text{0}.\text{0}\text{6}\text{2}\text{5} \end{array} \end{array}$$ (7)

A conventional 5% significance threshold is therefore unattainable in this finite assignment set, even if Taijing were ranked first. Given the single treated unit and finite donor pool, we characterize SCM uncertainty using exact in-space placebo rankings, following the established synthetic-control inference framework [17,26], rather than relying on conventional regression-style confidence intervals.

Accordingly, the SCM placebo exercise is interpreted as a finite-sample rank diagnostic of the relative extremeness of Taijing’s post-treatment divergence rather than as standalone evidence of statistical significance. Inferential precision is assessed separately using the baseline SDID estimates with placebo-based standard errors, confidence intervals, and p-values, together with the matching-weighted TWFE estimates using full-procedure bootstrap confidence intervals that account for generated-weight uncertainty. Given the small number of county clusters, Webb wild-cluster bootstrap p-values are additionally used as a conservative finite-sample check for the TWFE estimates. The three approaches therefore provide complementary inferential perspectives based on the same underlying panel: SCM emphasizes counterfactual fit and relative post-treatment divergence, SDID adds placebo-based uncertainty estimates under a different weighting procedure, and matching-weighted TWFE provides stage-specific estimates under alternative uncertainty corrections.

### 4.5 Robustness and extension tests

#### 4.5.1 Donor-pool sensitivity.

To assess whether the SCM estimates for tourism revenue depend excessively on the composition of the donor pool, the study conducts a set of donor-pool sensitivity tests. Excluding Kaili—a county with a relatively strong economic and tourism base—reduces the donor pool from 15 to 14 units but leaves the pre-treatment RMSPE, post-treatment RMSPE, RMSPE ratio, and the 2022 and 2023–2024 estimates unchanged. This additional donor-pool check indicates that the baseline tourism-revenue SCM result is not driven by the inclusion of Kaili.

#### 4.5.2 Leave-one-out tests.

We conduct a complete leave-one-out exercise in which each of the 15 donor counties is excluded once from the formal non-nested revenue SCM while retaining the same predictor set, 2015–2021 fitting period, and 2022 treatment date. The estimated tourism-revenue effects remain positive in all 15 specifications for both the 2022 breakout stage and the 2023–2024 institutional-expansion stage. However, the magnitude of the estimates is more sensitive to the exclusion of the three positive-weight donors in the baseline synthetic control: Majiang (weight = 0.723), Cengong (0.223), and Liping (0.055). Across the 15 leave-one-out specifications, the estimated 2022 effect ranges from 23.33% to 34.02%, while the 2023–2024 average effect ranges from 80.55% to 101.62%. Thus, the leave-one-out analysis supports the directional robustness of the estimated revenue response, while indicating that its precise magnitude depends on the composition of the positive-weight donor set, particularly the two dominant donors, Majiang and Cengong. Selected leave-one-out results are reported in Table 4, with the complete results provided in S4 Table.

**Table 4:** Selected leave-one-out sensitivity of tourism-revenue SCM estimates.

Scenario	Donors	Pre-treatment RMSPE	Post-treatment RMSPE	RMSPE ratio	2022 effect (%)	2023–2024 average effect (%)
Baseline SCM	15	0.066	0.563	8.574	29.95	94.31
Exclude Majiang	14	0.122	0.528	4.337	30.71	85.54
Exclude Cengong	14	0.085	0.497	5.868	23.33	80.55
Exclude Liping	14	0.067	0.597	8.941	34.02	101.62

Effects are calculated as 100[exp(log gap)−1]. The 2023–2024 effect is based on the mean annual log gap before transformation. Complete leave-one-out results for all 15 donor counties are reported in S4 Table.

#### 4.5.3 Cross-method consistency.

Across the baseline SCM, SDID, and matching-weighted TWFE specifications, the point estimates are directionally consistent. For both tourism revenue and tourist visitors, all three estimators yield positive baseline post-treatment estimates, and the estimated effects for 2023–2024 are larger than those for the immediate 2022 breakout stage. The strength of statistical evidence nevertheless differs across estimators and outcomes, particularly for tourism revenue. The three estimators yield a common directional and stage-differentiated pattern, although effect magnitudes and statistical support vary across methods. Because the 2023–2024 baseline point estimates exceed the corresponding 2022 estimates across all three estimators, the baseline results are also consistent with H2, although the COVID-period sensitivity analysis below qualifies this pattern for tourism revenue.

#### 4.5.4 COVID-19-period sensitivity analysis.

The baseline specifications retain 2020–2021 as pre-treatment years because both precede the 2022 breakout of Village BA. However, pandemic-related tourism disruptions may have evolved differently across counties, potentially affecting the construction of the pre-treatment counterfactual. We therefore conduct an additional sensitivity analysis in which the COVID-affected years are excluded from estimator construction. For SCM, donor weights are estimated using only the 2015–2019 pre-COVID period, while 2020–2021 are retained as an untreated holdout period to diagnose extrapolation beyond the pre-COVID fitting window; treatment effects are then evaluated over 2022–2024. For SDID and matching-weighted TWFE, 2020–2021 are removed from all counties, and the matching weights in the TWFE specification are reconstructed using 2015–2019 information only. The resulting SDID and TWFE estimation sample contains 16 counties observed over eight years (N = 128).

The holdout diagnostics indicate that the SCM counterfactual deteriorates when the pre-COVID weights are extrapolated into the COVID-affected period, particularly for tourism revenue. For tourism revenue, the RMSPE increases from 0.007 during the 2015–2019 fitting period to 0.581 during the untreated 2020–2021 holdout period; the corresponding post-treatment RMSPE for 2022–2024 is 0.151. For tourist visitors, the fitting-period, holdout-period, and post-treatment RMSPE values are 0.056, 0.273, and 0.292, respectively. These diagnostics indicate that the pre-COVID SCM specification does not extrapolate smoothly into 2020–2021, especially for tourism revenue. Accordingly, the COVID-excluded SCM serves as a sensitivity stress test for the choice of fitting window.

Consistent with this qualification, the COVID-excluded estimates present a more cautious picture than the baseline results. When SCM is fitted using only the 2015–2019 period, the estimated tourism-revenue gap is negative in 2022 but modestly positive on average during 2023–2024, whereas the tourist-visitor gap remains positive but is attenuated. The COVID-excluded SDID estimates remain positive for both outcomes but are imprecisely estimated, with all 95% confidence intervals including zero. In the matching-weighted TWFE specification, the treatment coefficients remain positive for both outcomes and in both stages, but none is statistically distinguishable from zero under the more conservative 9,999-replication Webb wild-cluster bootstrap inference. The sensitivity results therefore indicate that tourist-visitor estimates retain greater directional stability than tourism-revenue estimates, although inferential support for both outcomes weakens substantially once the COVID-affected years are excluded. Overall, the sensitivity analysis indicates that the estimated tourism-revenue response is more dependent on the construction of the pre-treatment counterfactual than the tourist-visitor response. In particular, the magnitude and stage-specific direction of the revenue estimate vary depending on whether the unusual 2020–2021 tourism trajectories are allowed to inform the counterfactual. The sensitivity estimates are reported in Table 5, with the corresponding SCM fitting- and holdout-period diagnostics provided in S5 Table.

**Table 5:** Sensitivity of treatment-effect estimates to the COVID-affected pre-treatment years.

Outcome	Period	SCM effect (%)	SDID ATT (SE)	95% CI	SDID p-value	Weighted TWFE β (SE)	Webb p-value
Tourism revenue	2022	−21.48	0.235 (0.475)	[−0.695, 1.166]	0.620	0.224(0.177)	0.318
Tourism revenue	2023–2024	6.93	0.585 (0.493)	[−0.382, 1.551]	0.236	0.525(0.206)	0.110
Tourist visitors	2022	3.43	0.217 (0.398)	[−0.562, 0.997]	0.584	0.286(0.117)	0.211
Tourist visitors	2023–2024	42.34	0.491 (0.477)	[−0.444, 1.426]	0.303	0.462(0.184)	0.148

SDID standard errors and p-values are based on 1,000 placebo replications and normal-approximation inference. Weighted-TWFE coefficients are accompanied by conventional county-clustered standard errors; p-values are based on 9,999-replication Webb wild-cluster bootstrap inference.

#### 4.5.5 Level-outcome sensitivity.

Because Taijing entered the treatment period from a comparatively low tourism-revenue base, the relatively large percentage effects obtained from the log specifications may partly reflect the proportional nature of the outcome measure. During the 2015–2021 pre-treatment period, Taijing’s average annual tourism revenue was approximately RMB 1.305 billion, compared with RMB 4.690 billion across the donor counties, meaning that Taijing’s pre-treatment revenue base was only about 27.8% of the donor-county average. To assess whether the positive post-treatment pattern is driven primarily by the logarithmic transformation or this relatively low initial base, we re-estimate the counterfactual models using tourism revenue in levels.

The level-based SCM continues to yield positive post-treatment absolute gaps. Relative to its synthetic counterfactual, Taijing’s tourism revenue is higher by approximately RMB 0.474 billion in 2022, RMB 1.731 billion in 2023, and RMB 2.259 billion in 2024. Because the SCM is re-estimated directly using tourism revenue in levels, these absolute gaps need not coincide exactly with the back-transformed monetary differences from the baseline log-SCM specification. These absolute differences indicate that the positive post-treatment divergence is not mechanically generated by expressing tourism revenue in logarithms. However, the corresponding level-based SDID estimates are considerably less precise. The estimated ATT is RMB 0.429 billion in 2022 (SE = 0.541 billion, 95% CI [−0.632, 1.491], p = 0.428) and RMB 1.320 billion on average during 2023–2024 (SE = 1.363 billion, 95% CI [−1.352, 3.991], p = 0.333). Both confidence intervals include zero. Detailed level-outcome SDID estimates are reported in S6 Table.

Viewed alongside the baseline log specifications, the level-outcome analysis places the relatively large proportional estimates in context. Their magnitude may partly reflect Taijing’s comparatively low pre-treatment tourism-revenue base, while the positive level-based SCM gaps indicate that the post-treatment divergence also corresponds to substantial absolute revenue differences.

## 5. Discussion

### 5.1 Core findings and theoretical dialogue: Counterfactual evidence on county-level tourism responses to a small event

This study uses the rapid expansion of Village BA in Taijing County as a quasi-experimental setting and applies a complementary identification strategy based on SCM, SDID, and matching-weighted TWFE to evaluate county-level tourism responses associated with the broader Village BA-centered expansion. After accounting for common trends and macro-level shocks as far as possible, the baseline evidence shows positive post-breakout deviations in both tourism revenue and tourist visitors. More importantly, the two tourism outcomes displayed different response patterns across stages. During the 2022 breakout stage, tourist-visitor estimates were comparatively more directionally stable, while tourism-revenue effects became more pronounced during the 2023–2024 institutional-expansion stage. The later-stage revenue response is comparatively precise in the baseline SDID and full-procedure bootstrap results, whereas tourist-visitor estimates retain greater directional stability across sensitivity specifications. This stage-differentiated pattern is broadly consistent with a relative “flows first, returns later” interpretation.

These findings contribute to literature in three ways. First, this study moves beyond the limitations of micro-level evaluation and establishes a county-level counterfactual benchmark for assessing the tourism effects of small-scale sport events. Recent reviews document considerable variation in the methods used to assess the economic and tourism outcomes of sports events [32]. Distinguishing additional economic activity from substitution, crowding-out, and leakage remains an important methodological challenge, particularly when translating visitor expenditure into estimates of wider economic impact [33]. Responding to Baade and Matheson’s (2016) call for counterfactual approaches in event evaluation [4], this study uses a data-driven synthetic Taijiang to account for broader post-pandemic recovery trends as far as possible within the available research design. In doing so, it provides county-level macro evidence of positive tourism gains associated with the broader Village BA-centered expansion beyond the initial publicity shock, although the COVID-period sensitivity analysis indicates that the estimated tourism-revenue effect remains sensitive to the construction of the pre-treatment counterfactual.

Second, by drawing on evidence from rural China, this study helps refine the boundary conditions of the conventional “mega-event syndrome.” Existing scholarship has long cautioned that mega-events often fall into a “white elephant” trap because of heavy infrastructure costs and capital leakage [34]. By contrast, the Village BA model relies more heavily on existing public facilities, community organization, local cultural resources, and social networks, which is broadly consistent with the endogenous-development logic emphasized by the RSLG framework. The positive tourism response observed in Taijing therefore provides context-specific evidence consistent with the proposition that locally embedded small-scale events may exhibit greater economic resilience than capital-intensive mega-events.

Third, this study reveals a clear stage-differentiated pattern in the evolution of tourism outcomes. Across the baseline methods, tourist-visitor estimates are comparatively stable during the 2022 breakout stage, while tourism-revenue effects become more pronounced during 2023–2024. This suggests that the tourism response did not simply dissipate after the initial publicity shock; instead, the later-stage estimates remained substantial, particularly for tourism revenue. The contrast between an early visitor-flow response and stronger later-stage revenue effects can be interpreted in light of research linking event attachment to place attachment and loyalty [23], and research emphasizing the strategic integration of events with destination products and services [35]. These perspectives offer plausible explanations for the observed pattern, although the present aggregate data do not directly identify the underlying mechanisms. This interpretation also highlights the importance of viewing the tourism consequences of small-scale rural events as an evolving process rather than as a one-off publicity effect.

### 5.2 Potential mechanism mapping: From statistical curves to psychological, behavioral, and structural leverage

The observed dynamic pattern can be interpreted through three interrelated layers of potential mechanisms—psychological, behavioral, and structural. These mechanisms provide a theory-grounded framework for understanding how event attention may be translated into broader tourism activity. Small-scale rural sport events have emerged as novel vehicles for stimulating integrated consumption across agriculture, culture, and tourism [36]. From the dual perspective of the event and the place, their distinctive appeal lies in cultural authenticity. Unlike highly commercialized professional leagues, the embeddedness of “Village BA” in Miao intangible cultural heritage and interactive rural practices may create conditions for deeply immersive emotional experiences. As suggested by previous research, affective attachment rooted in a strong sense of cultural authenticity may extend beyond the event itself, producing a spillover effect whereby visitors transfer their affection for the event to the host destination and develop stronger place attachment [37]. Previous research further suggests that such psychological translation may strengthen visitors’ willingness to return beyond the event period [38]. Such a psychological translation therefore provides one plausible explanation for the persistence of the observed tourism response after the initial event shock.

At the behavioral level, reputation accumulation and lower revisit costs provide a second possible interpretation. As the event accumulates brand equity, social media sharing and electronic word-of-mouth generated by early visitors increasingly become important information sources for subsequent potential tourists, thereby reducing search costs and perceived risk while reinforcing destination image and visit intention [39,40]. Existing tourism research further suggests that destination loyalty and repeat visitation can contribute to more stable tourism demand [41], while repeat tourists may exhibit different patterns of stay and activity from first-time visitors [42]. Given the documented positive—and potentially nonlinear—relationship between length of stay and total tourism expenditure [43], repeat visitation could, in principle, contribute to higher tourism spending through a stay–consumption channel. However, the county-level aggregate data used here do not contain individual visitor histories, length-of-stay information, or per-capita expenditure measures. Accordingly, the observed aggregate revenue pattern may reflect changes in visitor volume, spending per visitor, repeat visitation, or some combination of these channels.

At the structural level, the activation of event-combination leverage provides a third possible interpretation. Sport events may function less as self-sustaining engines of growth than as seed capital for attention and visitation, while the extent to which such attention is translated into broader destination consumption depends partly on operational and coordinated leverage strategies deployed before, during, and after the event [1,35]. This logic has been repeatedly emphasized in the literature on event leverage and event portfolios, which highlights the importance of bundling event experiences with accommodation, food and beverage services, cultural experiences, local products, and tourism routes in order to enhance local economic retention [28]. From an event-leverage perspective, the expansion of Village BA can be interpreted as involving broader forms of local coordination and destination-level resource integration. Such processes are consistent with the event-leverage literature, which emphasizes the importance of local stakeholder participation and business engagement in translating event-generated attention into broader local economic benefits [44]. In the case of Village BA, such coordination could plausibly strengthen the local capacity to connect event-generated attention with broader tourism consumption and thereby help explain the more pronounced tourism-revenue pattern observed during the later institutional-expansion stage. This interpretation also accords with prior research highlighting product development, destination bundling, and mechanisms of local value capture as important means of extending the benefits of one-off events [45].

Taken together, the psychological, behavioral, and structural layers provide a coherent framework for interpreting the observed tourism dynamics. From this perspective, the relative “flows first, returns later” pattern may reflect a gradual process in which event-generated attention is reflected in visitor activity and, over time, becomes connected with broader tourism consumption and local value capture, while the specific micro-level pathways underlying this process remain to be examined with more disaggregated visitor and expenditure data.

### 5.3 Identification boundaries and limitations

Despite the use of complementary identification strategies, the causal interpretation should remain cautious. First, nationwide tourism recovery after 2022 complicates the attribution of post-treatment tourism changes to Village BA. Counterfactual construction, fixed effects, covariate adjustment, and the COVID-period sensitivity analysis help account for this broader recovery process, although the tourism-revenue estimates remain sensitive to the construction of the pre-treatment counterfactual.

Second, the expansion of Village BA coincided with additional fiscal support, infrastructure improvements, media exposure, and other contemporaneous developments. These factors cannot be separately identified from the event itself within the present research design. Rather than treating them as exogenous confounders that can be cleanly removed, they are more appropriately understood as part of the broader event-centered treatment bundle surrounding the expansion of Village BA. Accordingly, the estimated post-2022 effects should be interpreted as the tourism effects associated with this broader bundle rather than as the isolated marginal effect of the tournament alone.

Third, Village BA may have generated spatial spillovers through visitor diversion, diffusion, or complementary tourism activity in surrounding counties. If such spillovers affected donor counties, the stable-unit-treatment-value assumption underlying the counterfactual design may be imperfectly satisfied. Although in-space placebo exercises, RMSPE-ratio diagnostics, donor-pool sensitivity tests, and leave-one-out analyses provide useful information on the stability of the estimated counterfactual, they cannot directly identify the geographic extent or direction of these spillovers. Future research could therefore combine higher-frequency tourism data with spatial econometric or network-based approaches to examine the geographic diffusion of event-related tourism effects more systematically.

### 5.4 Policy implications: From replicating the tournament’s surface form to replicating its leverage logic

The extraordinary success of Village BA has already inspired imitation across China. Yet the county-level evidence and theory-grounded mechanism discussion presented here suggest that simply copying the basketball format is unlikely to reproduce the same tourism outcomes. Two implications follow.

First, the findings suggest that rural event development should draw on locally distinctive sporting traditions, cultural resources, and community participation. Policy efforts should also connect event experiences with accommodation, food services, cultural heritage, and agricultural products through coordination among organizers, local businesses, and communities. These principles provide more useful guidance than simply reproducing the basketball format.

Second, the differences observed across stages further suggest that evaluation should extend beyond the initial publicity surge. Policymakers should monitor visitor numbers and tourism revenue separately over time and support organizational capacity and destination-service coordination, while recognizing that early tourism gains do not guarantee sustained returns.

Taken together, the study contributes a temporal perspective to the event-leverage debate by distinguishing visitor attraction from the realization of tourism revenue at the county level, without treating the underlying conversion mechanisms as empirically established.

## Supporting information

S1 Table SCM predictor V-weights. (RTF)

S2 Table Pre-treatment balance before and after matching. (RTF)

S3 Table Absolute monetary context for the baseline log-SCM estimates. (RTF)

S4 Table Complete leave-one-out sensitivity analysis for tourism-revenue SCM. (RTF)

S5 Table COVID-period SCM fitting, untreated holdout, and post-treatment diagnostics. (RTF)

S6 Table Level-outcome SDID sensitivity analysis for tourism revenue. (RTF)

S7 Table Full matching-weighted TWFE regression results. (RTF)

S1 File County-year analysis dataset used for the empirical analyses and Stata code. (ZIP)
