# Supplementary material for: Small events, big impact: Can rural sports event IP generate county-level tourism dividends? Evidence from “Village BA” in Taijiang County, China
Source: PLoS One. 2026 Nov 11;21(11):e0348851. doi: 10.1371/journal.pone.0348851 (PMC13754218; doi:10.1371/journal.pone.0348851)
Supplement: Supplementary file 1 [file pone.0348851.s001.rtf]

S1 Table. SCM predictor V-weights.
Outcome and predictor	Normalized V-weight	
Tourism revenue: Log tourist visitors	0.000000	
Tourism revenue: Log per-capita GDP	0.000000	
Tourism revenue: Log resident population	0.000000	
Tourism revenue: Log budget revenue	0.000000	
Tourism revenue: Log budget expenditure	0.000000	
Tourism revenue: Log tourism revenue, 2015	0.132907	
Tourism revenue: Log tourism revenue, 2016	0.170855	
Tourism revenue: Log tourism revenue, 2017	0.245931	
Tourism revenue: Log tourism revenue, 2018	0.148681	
Tourism revenue: Log tourism revenue, 2019	0.145565	
Tourism revenue: Log tourism revenue, 2020	0.047438	
Tourism revenue: Log tourism revenue, 2021	0.108622	
Tourist visitors: Log tourism revenue	0.000000	
Tourist visitors: Log per-capita GDP	0.000000	
Tourist visitors: Log resident population	0.000000	
Tourist visitors: Log budget revenue	0.000000	
Tourist visitors: Log budget expenditure	0.000000	
Tourist visitors: Log tourist visitors, 2015	0.130207	
Tourist visitors: Log tourist visitors, 2016	0.179451	
Tourist visitors: Log tourist visitors, 2017	0.173299	
Tourist visitors: Log tourist visitors, 2018	0.167446	
Tourist visitors: Log tourist visitors, 2019	0.155615	
Tourist visitors: Log tourist visitors, 2020	0.057848	
Tourist visitors: Log tourist visitors, 2021	0.136134	
Note:V-weights are from the formal non-nested SCM. Values close to zero indicate negligible relative predictor weight.
