# Supplementary material for: Small events, big impact: Can rural sports event IP generate county-level tourism dividends? Evidence from “Village BA” in Taijiang County, China
Source: PLoS One. 2026 Nov 11;21(11):e0348851. doi: 10.1371/journal.pone.0348851 (PMC13754218; doi:10.1371/journal.pone.0348851)
Supplement: Supplementary file 2 [file pone.0348851.s002.rtf]

S2 Table. Pre-treatment balance before and after matching.
Pre-treatment characteristic	Taijiang	Unweighted donor mean	Weighted donor mean	Standardized gap before	Standardized gap after	Reduction (%)	
Mean log tourism revenue	2.492	3.182	2.767	0.756	0.302	60.1	
Trend log tourism revenue	0.162	0.151	0.154	0.116	0.077	33.7	
Mean log tourist visitors	5.104	5.786	5.434	0.848	0.410	51.6	
Trend log tourist visitors	0.133	0.111	0.116	0.324	0.257	20.8	
Mean log percapita GDP	10.365	10.181	10.235	1.138	0.802	29.5	
Mean log resident population	2.480	3.040	2.757	1.140	0.564	50.6	
Mean log budget revenue	9.990	10.285	9.978	0.492	0.019	96.1	
Mean log budget expenditure	11.989	12.306	12.132	0.937	0.422	54.9	
Note: Standardized gaps use the unweighted donor standard deviation. Matching uses the exponential kernel with lambda = 1.
