# Supplementary material for: Small events, big impact: Can rural sports event IP generate county-level tourism dividends? Evidence from “Village BA” in Taijiang County, China
Source: PLoS One. 2026 Nov 11;21(11):e0348851. doi: 10.1371/journal.pone.0348851 (PMC13754218; doi:10.1371/journal.pone.0348851)
Supplement: Supplementary file 3 [file pone.0348851.s003.rtf]

S3 Table. Absolute monetary context for the baseline log-SCM estimates 
Year	Actual revenue (RMB billion)	Synthetic revenue (RMB billion)	Absolute gap (RMB billion)	Log gap	Effect (%)	
2022	1.712	1.317	0.395	0.262	29.95	
2023	3.333	1.714	1.619	0.665	94.42	
2024	4.366	2.248	2.118	0.664	94.19	
Note: Effects equal 100*[exp(log gap)-1]. Monetary amounts are reported in RMB billion.
