# Supplementary material for: Small events, big impact: Can rural sports event IP generate county-level tourism dividends? Evidence from “Village BA” in Taijiang County, China
Source: PLoS One. 2026 Nov 11;21(11):e0348851. doi: 10.1371/journal.pone.0348851 (PMC13754218; doi:10.1371/journal.pone.0348851)
Supplement: Supplementary file 4 [file pone.0348851.s004.rtf]

S4 Table. Complete leave-one-out sensitivity analysis for tourism-revenue SCM.
Excluded donor	Baseline weight	Pre-treatment RMSPE	Post-treatment RMSPE	RMSPE ratio	2022 effect (%)	2023-2024 effect (%)	
Majiang	0.722	0.122	0.528	4.337	30.71	85.54	
Cengong	0.223	0.085	0.497	5.868	23.33	80.55	
Liping	0.055	0.067	0.597	8.941	34.02	101.62	
Sansui	0.000	0.066	0.563	8.574	29.95	94.31	
Danzhai	0.000	0.066	0.563	8.574	29.95	94.31	
Congjiang	0.000	0.066	0.563	8.574	29.95	94.31	
Kaili	0.000	0.066	0.563	8.574	29.95	94.31	
Jianhe	0.000	0.066	0.563	8.574	29.95	94.31	
Tianzhu	0.000	0.066	0.563	8.574	29.95	94.31	
Shibing	0.000	0.066	0.563	8.574	29.95	94.31	
Rongjiang	0.000	0.066	0.563	8.574	29.95	94.31	
Jinping	0.000	0.066	0.563	8.574	29.95	94.31	
Zhenyuan	0.000	0.066	0.563	8.574	29.95	94.31	
Leishan	0.000	0.066	0.563	8.574	29.95	94.31	
Huangping	0.000	0.066	0.563	8.574	29.95	94.31	
Note: Each row excludes the named donor from the formal non-nested revenue SCM. All specifications retain the 2015-2021 fitting period and 2022 treatment date. Effects equal 100*[exp(log gap)-1].
