# Supplementary material for: Small events, big impact: Can rural sports event IP generate county-level tourism dividends? Evidence from “Village BA” in Taijiang County, China
Source: PLoS One. 2026 Nov 11;21(11):e0348851. doi: 10.1371/journal.pone.0348851 (PMC13754218; doi:10.1371/journal.pone.0348851)
Supplement: Supplementary file 5 [file pone.0348851.s005.rtf]

S5 Table. COVID-period SCM fitting, untreated holdout, and post-treatment diagnostics.
Outcome	2015-2019 fitting RMSPE	2020-2021 untreated holdout RMSPE	2022-2024 post-treatment RMSPE	2022 log gap	2022 effect (%)	2023-2024 average log gap	2023-2024 effect (%)	
Tourism revenue	0.007	0.581	0.151	-0.242	-21.48	0.067	6.93	
Tourist visitors	0.056	0.273	0.292	0.034	3.43	0.353	42.34	
Note: SCM weights are estimated using 2015-2019 only. The 2020-2021 COVID-affected years are an untreated holdout period. Effects equal 100*[exp(log gap)-1].
