# Supplementary material for: Small events, big impact: Can rural sports event IP generate county-level tourism dividends? Evidence from “Village BA” in Taijiang County, China
Source: PLoS One. 2026 Nov 11;21(11):e0348851. doi: 10.1371/journal.pone.0348851 (PMC13754218; doi:10.1371/journal.pone.0348851)
Supplement: Supplementary file 6 [file pone.0348851.s006.rtf]

S6 Table. Level-outcome SDID sensitivity analysis for tourism revenue.
Period	ATT (RMB billion)	Placebo SE (RMB billion)	95% CI lower
	95% CI upper	p-value	
2022	0.429	0.541	-0.632	1.491	0.428	
2023-2024	1.320	1.363	-1.352	3.991	0.333	
Note: Placebo standard errors use 1,000 replications. Confidence intervals and p-values use normal approximation based on the placebo standard errors.
