# Supplementary material for: Small events, big impact: Can rural sports event IP generate county-level tourism dividends? Evidence from “Village BA” in Taijiang County, China
Source: PLoS One. 2026 Nov 11;21(11):e0348851. doi: 10.1371/journal.pone.0348851 (PMC13754218; doi:10.1371/journal.pone.0348851)
Supplement: Supplementary file 7 [file pone.0348851.s007.rtf]

S7 Table. Full matching-weighted TWFE regression results.
Panel A: Baseline (2015-2024).
	Coefficient	Clustered SE	95% CI lower	95% CI upper	Clustered p-value	Webb p-value	N	R-squared	Adjusted R-squared	
Log tourism revenue: Treatment x 2022	0.186	0.133	-0.098	0.469	0.183	0.320	160	0.859	0.826	
Log tourism revenue: Treatment x Post 2023 2024	0.516	0.153	0.191	0.841	0.004	0.054	160	0.859	0.826	
Log tourism revenue: Log per capita GDP	0.490	0.372	-0.303	1.283	0.208	.	160	0.859	0.826	
Log tourism revenue: Log resident population	1.538	1.843	-2.392	5.467	0.417	.	160	0.859	0.826	
Log tourism revenue: Log budget revenue	-0.396	0.315	-1.068	0.275	0.228	.	160	0.859	0.826	
Log tourism revenue: Log budget expenditure	0.393	0.442	-0.549	1.335	0.388	.	160	0.859	0.826	
Log tourist visitors: Treatment x 2022	0.209	0.063	0.073	0.344	0.005	0.058	160	0.911	0.890	
Log tourist visitors: Treatment x Post 2023 2024	0.457	0.110	0.222	0.691	0.001	0.030	160	0.911	0.890	
Log tourist visitors: Log per capita GDP	0.505	0.448	-0.449	1.460	0.277	.	160	0.911	0.890	
Log tourist visitors: Log resident population	1.474	1.752	-2.260	5.209	0.413	.	160	0.911	0.890	
Log tourist visitors: Log budget revenue	-0.089	0.119	-0.342	0.164	0.465	.	160	0.911	0.890	
Log tourist visitors: Log budget expenditure	0.189	0.296	-0.442	0.821	0.532	.	160	0.911	0.890	

Panel B: COVID-excluded (2015-2019 and 2022-2024).
	Coefficient	Clustered SE	95% CI lower	95% CI upper	Clustered p-value	Webb p-value	N	R-squared	Adjusted R-squared	
Log tourism revenue: Treatment x 2022	0.224	0.177	-0.153	0.601	0.224	0.318	128	0.895	0.865	
Log tourism revenue: Treatment x Post 2023 2024	0.525	0.206	0.086	0.963	0.022	0.110	128	0.895	0.865	
Log tourism revenue: Log per capita GDP	0.735	0.508	-0.349	1.818	0.169	.	128	0.895	0.865	
Log tourism revenue: Log resident population	1.677	1.379	-1.262	4.616	0.243	.	128	0.895	0.865	
Log tourism revenue: Log budget revenue	-0.387	0.338	-1.107	0.334	0.270	.	128	0.895	0.865	
Log tourism revenue: Log budget expenditure	0.554	0.425	-0.352	1.461	0.212	.	128	0.895	0.865	
Log tourist visitors: Treatment x 2022	0.286	0.117	0.038	0.534	0.027	0.211	128	0.933	0.914	
Log tourist visitors: Treatment x Post 2023 2024	0.462	0.184	0.071	0.854	0.024	0.148	128	0.933	0.914	
Log tourist visitors: Log per capita GDP	0.881	0.858	-0.947	2.709	0.321	.	128	0.933	0.914	
Log tourist visitors: Log resident population	2.507	1.745	-1.211	6.225	0.171	.	128	0.933	0.914	
Log tourist visitors: Log budget revenue	-0.059	0.175	-0.431	0.313	0.741	.	128	0.933	0.914	
Log tourist visitors: Log budget expenditure	0.301	0.329	-0.400	1.003	0.375	.	128	0.933	0.914	
Note: County fixed effects: Yes. Year fixed effects: Yes Standard errors are clustered by county. Webb wild-cluster bootstrap p-values use 9999 replications, seed 20260820,and are reported only for treatment coefficients. Panel A weights use 2015-2021 features; Panel B weights use 2015-2019 features and excludes 2020-2021 for every county.
